# Intermediary Metabolite Precursor Dimethyl-2-Ketoglutarate Stabilizes Hypoxia-Inducible Factor-1α by Inhibiting Prolyl-4-Hydroxylase PHD2

**DOI:** 10.1371/journal.pone.0113865

**Published:** 2014-11-24

**Authors:** Peifeng Hou, Ching-Ying Kuo, Chun-Ting Cheng, Jing-Ping Liou, David K. Ann, Qiang Chen

**Affiliations:** 1 Department of Molecular Pharmacology, Beckman Research Institute, City of Hope, Duarte, California, United States of America; 2 Department of Oncology, Fujian Medical University Union Hospital, Fuzhou, Fujian, China; 3 Irell & Manella Graduate School of Biological Sciences, Beckman Research Institute, City of Hope, Duarte, California, United States of America; 4 Fujian Key Laboratory of Translational Cancer Medicine, Fuzhou, Fujian, China; 5 School of Pharmacy, College of Pharmacy, Taipei Medical University, Taipei, Taiwan; School of Medicine and Health Sciences, University of North Dakota, United States of America

## Abstract

Hypoxia-inducible factor 1α (HIF-1α), a major mediator of tumor physiology, is activated during tumor progression, and its abundance is correlated with therapeutic resistance in a broad range of solid tumors. The accumulation of HIF-1α is mainly caused by hypoxia or through the mutated succinate dehydrogenase A (SDHA) or fumarate hydratase (FH) expression to inhibit its degradation. However, its activation under normoxic conditions, termed pseudohypoxia, in cells without mutated SDHA or FH is not well documented. Here, we show that dimethyl-2-ketoglutarate (DKG), a cell membrane-permeable precursor of a key metabolic intermediate, α-ketoglutarate (α-KG), known for its ability to rescue glutamine deficiency, transiently stabilized HIF-1α by inhibiting activity of the HIF prolyl hydroxylase domain-containing protein, PHD2. Consequently, prolonged DKG-treatment under normoxia elevated HIF-1α abundance and up-regulated the expression of its downstream target genes, thereby inducing a pseudohypoxic condition. This HIF-1α stabilization phenotype is similar to that from treatment of cells with desferrioxamine (DFO), an iron chelator, or dimethyloxalyglycine (DMOG), an established PHD inhibitor, but was not recapitulated with other α-KG analogues, such as Octyl-2KG, MPTOM001 and MPTOM002. Our study is the first example of an α-KG precursor to increase HIF-1α abundance and activity. We propose that DKG acts as a potent HIF-1α activator, highlighting the potential use of DKG to investigate the contribution of PHD2-HIF-1α pathway to tumor biology.

## Introduction

Hypoxia-inducible factor-1α (HIF-1α) is a key transcription factor, and its overexpression is linked to a myriad of pathological consequences in many types of cancer, including breast, ovarian, renal carcinoma, glioblastoma and leiomyoma ([1] [2] and references therein). The stability of the HIF-1α protein is tightly controlled by O_2_ availability. Under normoxia, both proline (Pro)402 and Pro564 located at the O_2_-dependent degradation (ODD) domain of HIF-1α are hydroxylated in the presence of ascorbate, alpha-ketoglutarate (α-KG) or Fe(II) by the prolyl hydroxylase domain (PHD)-containing proteins [3], [4]. The von Hippel-Lindau tumor suppressor (pVHL) E3 ligase complex recognizes HIF-1α with either proline hydroxylated, leading to HIF-1α ubiquitylation and degradation by the proteasome [5]. PHD2 is the most abundant HIF prolyl hydroxylase for HIF-1α degradation [6], [7]. Mutations of respective proline located within the N- and C-terminal ODD domain showed approximately equal amounts of increased stability and reduced up-regulation in hypoxia. If both prolines are substituted, HIF-1α shows further increase in stability and no induction in hypoxia [8], [9].

In cell culture, HIF-1α proline-hydroxylation is inhibited by lower O_2_ tension (below 6%, termed as hypoxia). Under hypoxic condition, the activity of PHD is restricted, thereby inhibiting HIF-1α hydroxylation and degradation [4]. Consequently, induced expression of HIF-1α downstream target genes, including *GLUT-1*, *PDK1 and CAIX*, provides survival advantages to tumor cells [10]. Alternatively, loss-of-function mutations in pVHL or the enzymes involved in the metabolism of PHD cofactors cause the accumulation of HIF-1α in cancers. For example, sporadic renal cell carcinoma with mutations or deletions of *VHL* gene exhibits elevated HIF-1α levels [11]. Succinate dehydrogenase (SDH) and fumarate hydratase (FH) are the enzymes that hydrolyze succinate and fumarate, respectively, to fuel the tricarboxylic acid (TCA) cycle. Mutations in SDH or FH are found in cancers and cause succinate or fumarate to accumulate and compete with α-KG for PHD binding, thereby inhibiting PHD and stabilizing HIF-1α [12], [13]. Mutations have also been identified in isocitrate dehydrogenase 1 (IDH1) that inhibit IDH1 catalytic activity in glioma, thereby reducing the production of α-KG, increasing HIF-1α and presumably, tumorigenesis [14]. Therefore, dysregulated cellular metabolome could up-regulate HIF-1α level and create a pseudohypoxic state under normoxia. Other factors also affect the amount of HIF-1α available for transcriptional activation. These include increased HIF-1α production by activated mTOR signaling [15] and VHL-independent degradation by heat shock protein 70 (Hsp70) and carboxyl terminus of HSP-interaction protein (CHIP) [16]. Moreover, the availability of nutrients, such as glutamine (Gln) and glucose (Glc), also regulates the translation of HIF-1α. It has been shown that the decreased levels of Gln or Glc inhibit the translation of HIF-1α, thereby decreasing the level of HIF-1α [17], suggesting that the fluctuation of metabolites could modulate HIF-1α activity and its downstream signaling to regulate cellular responses.

In the present study, we show that dimethyl-2-ketoglutarate (DKG), a precursor for α-KG, increases HIF-1α abundance and activity. We demonstrate that PHD2 is one of the potential targets for DKG to stabilize HIF-1α. Taken together, our studies identify DKG, unlike α-KG, acts to promote pseudohypoxia by promoting HIF-1α accumulation and function.

## Materials and Methods

### Cell lines and reagents

All cell lines were cultured in a humidified 5% CO_2_ incubator at 37°C. Human BC cell lines: MDA-MB-231, MCF7, human fibroblast cell line HS-5 [18] (purchased from ATCC CRL-11882) and HEK293 cells were all cultured in Dulbecco's modified Eagle's medium (DMEM) containing 10% fetal bovine serum (FBS) and penicillin (100 U/ml)-streptomycin (100 µg/ml). The human mammary epithelial cell line, MCF-10A [19] (a gift from Dr. Emily Wang, City of Hope, originally purchased from ATCC CRL-10317), was grown in DMEM supplemented with 5% horse serum, cholera toxin (0.1 µg/ml), insulin (10 µg/ml), hydrocortisone (0.5 µg/ml) and epidermal growth factor (20 ng/ml). Normal human dermal fibroblasts (NHDF) were cultured in minimum essential medium alpha (MEM-A) with antibiotics and 15% FBS. Hypoxic treatment was performed using an OxyCycler system (model C42; BioSpherix, Redfield, NY) set at 1% O_2_, according to the manufacturer's instructions. Dimethyl 2-ketoglutarate (DKG), desferrioxamine (DFO), dimethyloxalylglycine (DMOG) and cycloheximide (CHX) were purchased from Sigma. Octyl 2-ketoglutarate (Octyl-2KG) and its derivatives MPTOM001 and MPTOM002 were synthesized by Dr. Jing-Ping Liou (Taipei Medical University). MG132 was obtained from Calbiochem.

### Western blots and antibodies

For protein expression studies, whole cell lysates were extracted by SDS lysis buffer as previously described [20], [21], and supplemented with Complete protease inhibitor mixture (Roche Applied Science). Equal amounts of whole cell lysates were loaded, the proteins were separated by SDS-PAGE and immunoblotted with antibodies that recognized HIF-1α (BD Bioscience, 610958), HIF-2α (Novus Biologicals, NB100-122), phospho-p70S6K (Thr389, Cell Signaling, 9234), p70S6K (Cell Signaling, 9202), phospho-4EBP1 (Thr37/46, Cell Signaling, 2855), 4EBP1 (Cell Signaling, 9452), hydroxy-HIF1α (Pro564, Cell Signaling, 3434), CAIX (GeneTex, GTX70020). Anti-GAPDH (Santa Cruz Biotechnology, sc-25778) and anti-Actin (Millipore, 2020280) antibodies were used to assess equal protein loading. Immunoblots were visualized by an enhanced chemiluminescence detection kit (ECL-Plus, Amersham Pharmacia Biotech) and were imaged with a Versadoc 3000 Imaging System (Bio-Rad). Densitometric tracing were obtained and quantitated with Quantity One Software (Bio-Rad).

### Reverse transcription (RT) and quantitative PCR (qPCR)

Total RNA was extracted from cells using an RNeasy Mini Kit (Qiagen) according to manufacturer instructions. cDNA was synthesized from the total RNA using an iScript cDNA Synthesis Kit (Bio-Rad). Quantitative PCR analyses of targeted sequences were generated using the iTaq SYBR Green Supermix (Bio-Rad), a fraction of each cDNA sample, and gene-specific primer pairs: *HIF-1α*: 5′-CCACCTATGACCTGCTTGGT-3′ (Forward), 5′-TATCCAGGCTGTGTCGACTG-3′ (Reverse); *GLUT-1*: 5′- ATTGGCTCCGGTATCGTCAAC-3′ (Forward), 5′-GCTCAGATAGGACATCCAGGGTA-3′ (Reverse); *PDK1*: 5′- GAGAGCCACTATGGAACACCA-3′ (Forward), 5′-GGAGGTCTCAACACGAGGT-3′ (Reverse); *CAIX*: 5′- GTGCCTATGAGCAGTTGCTGTC-3′ (Forward), 5′-AAGTAGCGGCTGAAGTCAGAGG-3′ (Reverse); *p21*: 5′- TTTCTCTCGGCTCCCCATGT-3′ (Forward), 5′-GCTGTATATTCAGCATTGTGGG-3′ (Reverse); *GAPDH*: 5′- CCCCTTCATTGACCTCAACTA-3′ (Forward), 5′-CTCCTGGAAGATGGTGATGG-3′ (Reverse). PCR amplification and fluorescence were detected using a MyIQ real-time PCR detection system, and threshold cycles were determined by iCycler program (default setting). Fold induction was determined using the ΔΔC_T_ method normalized to GAPDH.

### Dual-luciferase reporter assay

The *p21-Luc* reporter construct was cloned as previously described [22], [23]. HA-HIF-1α-pcDNA3 was purchased from Addgene 18949 [24]. ODD-Luc-pcDNA3 was obtained from Addgene 18965 [25]. For the ODD-luciferase assay, HEK293 cells were transfected with ODD-Luc-pcDNA3 using Lipofectamine 2000 (Invitrogen) following the manufacturer's instructions. To evaluate p21 promoter activity, HEK293 cells were co-transfected with *p21-Luc* and HA-HIF-1α. A renilla luciferase control reporter pRL-TK was also co-transfected. Firefly luciferase activity was assayed with the Dual-Luciferase Reporter Assay System (Promega) and normalized against renilla luciferase activity.

### Small interfering RNA (siRNA) transfection and semi-quantitative nested RT-PCR

siRNAs that targeted PHD1, PHD2, PHD3 and a control siRNA were purchased from Santa Cruz Biotechnology. Cells were transfected using Lipofectamine RNAiMAX (Invitrogen) according to manufacturer's instructions. The efficiency of siRNA was determined by semi-quantitative nested RT-PCR of the relevant genes using gene-specific primer pairs purchased from Santa Cruz Biotechnology.

### Acid phosphatase (ACP) assay

Cell proliferation was measured by ACP assay. MDA-MB-231 cells pre-treated with DKG (10 mM, 7-day) were seeded at 5000 cells/well in the 96-well plate. Cells were treated with increasing doses (0–400 nM) of doxorubicin for 72-hour. To perform the ACP assay, cells were washed with PBS once and incubate with 4-Nitrophenyl phosphate disodium salt hexahydrate (pNPP) (7 mM in 0.1 M sodium acetate and 0.1% Triton X-100, pH 5.0) at 37°C for 30-min. Cells were lysed by adding 1 N NaOH and the absorbance was recorded at 410 nm using a SpectraMax M5 Multi-Mode Microplate Reader (Molecular Devices).

### Apoptosis analysis

Apoptosis was analyzed by Annexin V/Propidium iodide (PI) staining (BD Pharmingen) according to manufacturer's instructions. In brief, MDA-MB-231 cells were treated with DKG (10 mM, 24-hour), trypsinized, washed with PBS and re-suspended in binding buffer. Approximately 1×10^5^ cells were stained with Annexin V-FITC and PI, incubated at room temperature for 15-min, and analyzed by Accuri C6 Flow Cytometer (BD Biosciences).

### Statistical analyses

The data were analyzed using Microsoft Excel software. Each experiment was performed at least three times to obtain presented mean ± SD. Statistical significance was determined by two-tailed Student's *t*-test. *p*<0.05 is considered significant. _*_: *p*<0.05; _**_: *p*<0.01.

## Results

### DKG increases HIF-1α abundance under normoxia

**Figure 1:**
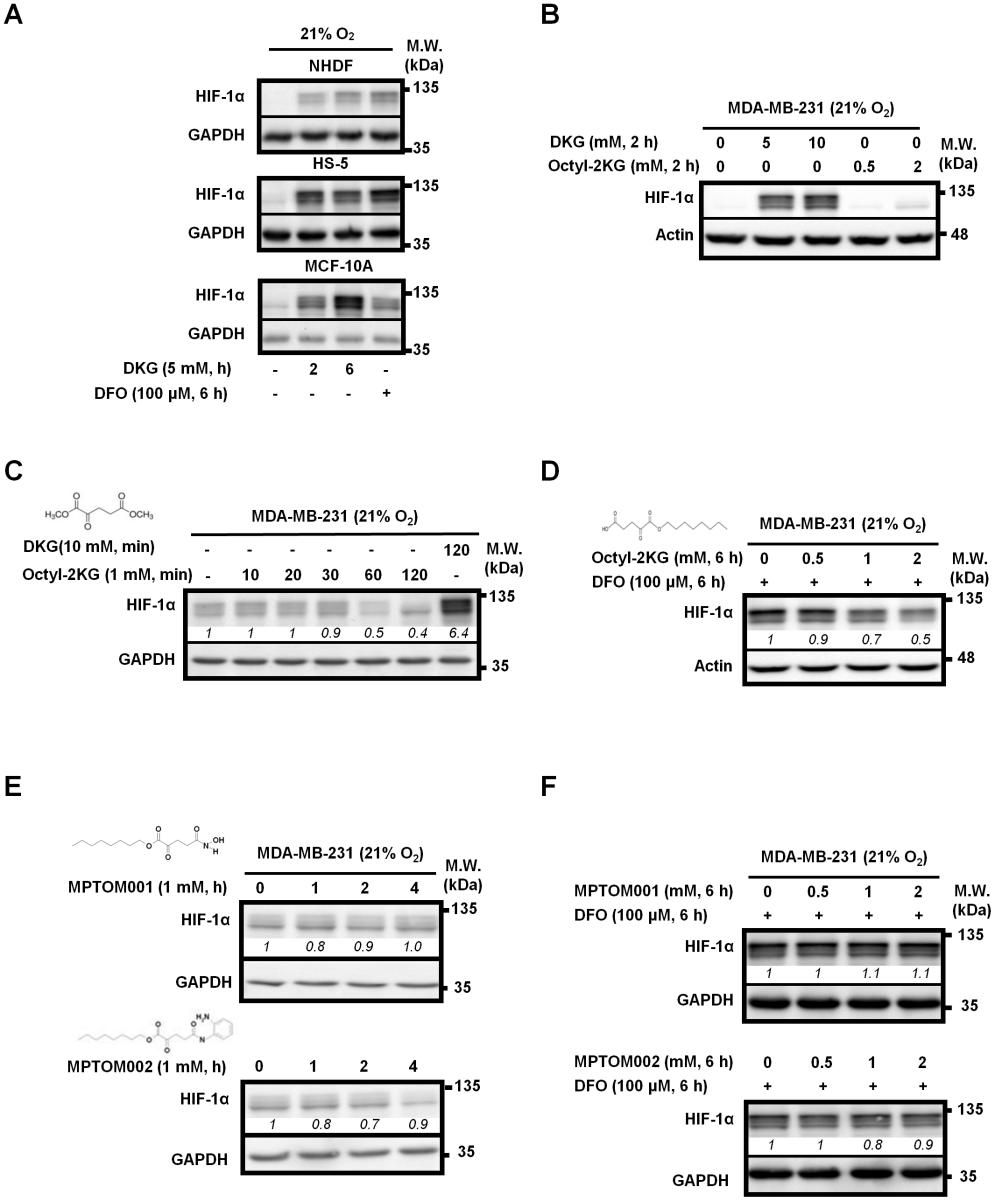
DKG promotes the HIF-1α accumulation in multiple cell lines under normoxia. (**A**) DKG elevates the steady-state HIF-1α abundance in non-cancerous cells. Human NHDF fibroblasts, stromal HS-5 cells and mammary epithelial MCF-10A cells were incubated with DKG under normoxia. The treatment with DFO (100 µM, 6-hour) to mimic hypoxia serves as a positive control. (**B**) DKG, but not Octyl-2KG, increases HIF-1α abundance in MDA-MB-231 cells under normoxia. MDA-MB-231 cells were treated with increasing doses of either DKG or Octyl-2KG for 2-hour. (**C**) Octyl-2KG decreases the steady-state HIF-1α protein abundance in a time-dependent manner. MDA-MB-231 cells were treated with Octyl-2KG (1 mM) for the indicated time periods. Cells treated with DKG (10 mM, 120-min) served as a positive control. (**D**) Octyl-2KG decreases HIF-1α protein level induced by DFO. MDA-MB-231 cells were treated with DFO (100 µM) and increasing doses of Octyl-2KG for 6-hour. (**E–F**) Octyl-2KG derivatives, MPTOM001 and MPTOM002, do not affect HIF-1α protein levels. MDA-MB-231 cells were treated with MPTOM001 or MPTOM002 in the absence (**E**) or presence (**F**) of DFO (100 µM). The chemical structures of DKG, Octyl-2KG, MPTOM001 and MPTOM002 are shown in (**C**, **D**, **E**). (**A–F**) Equal amount of whole cell lysates was analyzed by Western blots. GAPDH or Actin serves as a loading control. n = 3. A representative Western image from 3 independent experiments is shown. *Italic numbers* indicate the relative protein level after normalization with the level in the untreated cells.

To gain insight into the regulation of HIF-1α abundance by small molecules, we discovered that dimethyl-2-ketoglutarate (DKG), the precursor of α-ketoglutarate (α-KG) of tricarboxylic acid (TCA) cycle intermediate, elevated HIF-1α protein level, in human NHDF fibroblasts, marrow stromal HS-5 cells and mammary epithelial MCF-10A cells under normoxia (Fig. 1A). The extent of induction was comparable to that of desferrioxamine (DFO), an iron chelator known to stabilize HIF-1α [26]. Next, we assessed the effect of DKG and compared it, side by side, with Octyl-2KG on the regulation of HIF-1α abundance in breast cancer MDA-MB-231 cells. Octyl-2KG reportedly promotes hydroxylation of Pro-residues located at the HIF-1α ODD domain to down-regulate HIF-1α [14], [27]. As shown in Figs. 1B and 1C, HIF-1α protein abundance was notably elevated by DKG under normoxia. In contrast, no appreciable induction of HIF-1α was observed in cells treated with Octyl-2KG at various time points. Instead, the steady-state HIF-1α level was, consistent with previous reports [14], [27], down-regulated by 50% at 60-min post-treatment (Fig. 1C). Moreover, Octyl-2KG blocked the induction of HIF-1α by DFO (Fig. 1D). We further modified Octyl-2KG to generate two additional derivatives, but we did not observe any change in HIF-1α levels in cells treated with these two compounds under normoxia (Fig. 1E) or DFO-mimicked hypoxia (Fig. 1F). Altogether, the results suggest that unlike Octyl-2KG, DKG acts to increases HIF-1α abundance rather than to promote its degradation.

### DKG blocks HIF-1α degradation

**Figure 2:**
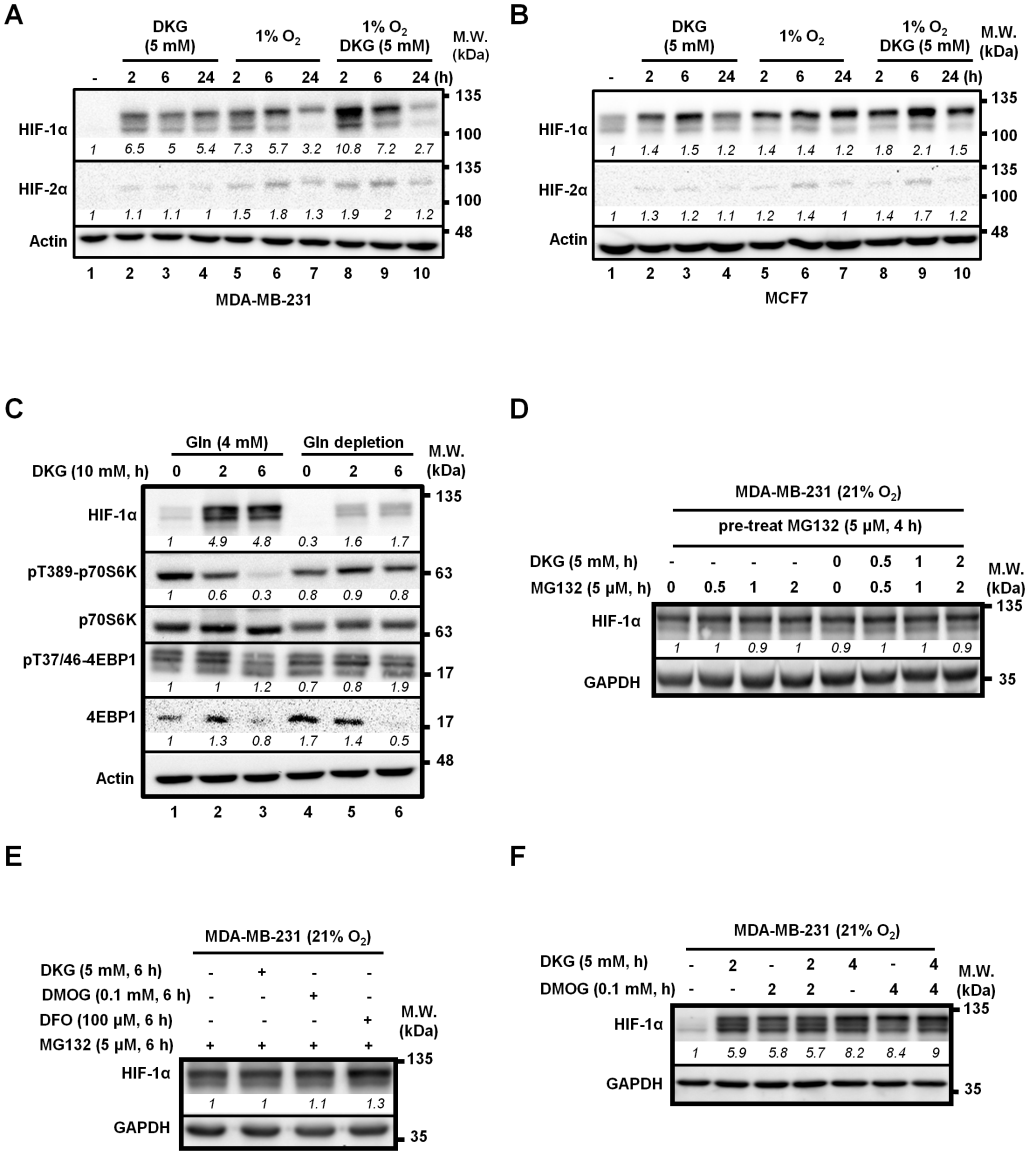
DKG blocks HIF-1α degradation, rather than inducing its transcription or translation. (**A, B**) DKG increases HIF-1α abundance in both normoxic and hypoxic conditions. MDA-MB-231 (A) and MCF7 (B) cells were cultured in normoxia (21% O_2_) or hypoxia (1% O_2_) for 2-, 6- or 24-hour in the presence or absence of DKG (5 mM). (**C**) DKG reduces mTOR signaling. MDA-MB-231 cells were treated with DKG in the presence or absence of Gln. The signals of pT389-p70S6K and pT37/46-4EBP1 were used to as surrogate markers for mTOR activation. (**D**) DKG fails to further elevate HIF-1α protein abundance in cells pre-treated with MG132. MDA-MB-231 cells were pre-treated with a proteasome inhibitor, MG132 (5 µM, 4-hour) to block the proteasomal degradation. The cells were then continuously treated with MG132 and vehicle or DKG to assess HIF-1α abundance. (**E**) DKG, like DMOG or DFO, converges on proteasome-mediated degradation of HIF-1α. MDA-MB-231 cells treated with MG132 were co-treated with DKG (5 mM), DMOG (0.1 mM) or DFO (100 µM) for 6-hour and assessed for HIF-1α abundance. (**F**) Inhibiting PHD does not further increase the level of HIF-1α protein induced by DKG. MDA-MB-231 cells were treated with DKG (5 mM), DMOG (0.1 mM) or both for the indicated time periods under normoxia. (**E**–**F**) Numbers in *italic* represent the relative levels of HIF-1α protein. The level in untreated cells was set to 1. (**A**, **C**–**F**) A representative Western image from 3 independent experiments is shown. *Italic numbers* indicate the relative protein level after normalization with the level in the untreated cells set as 1.

To explore how DKG elevated HIF-1α abundance, we sought to determine if DKG increased the steady-state *HIF-1α* mRNA level, and found that the *HIF-1α* mRNA abundance in MDA-MB-231 cells was not increased at all following DKG-treatment (Fig. S1A). Given that both HIF-1α and HIF-2α accumulate upon hypoxic exposure [28], we next checked whether DKG increased HIF-1α or HIF-2α abundance under hypoxia. Clearly, the effects by DKG and that by hypoxia on HIF-1α abundance were not mutually exclusive in both MDA-MB-231 (Fig. 2A) and MCF7 (Fig. 2B) cells, albeit with different kinetics. As shown in Figs. 2A and 2B, the combined treatment modestly promoted HIF-1α accumulation over individual treatments at 2- and 6-hour post-exposure (*lanes* 8–10 versus *lanes* 2–4 and 5–7). Along the same line, PHDs (mainly PHD2) also target Pro531 of HIF-2α for hydroxylation, thereby regulating HIF-2α stability [3], [4]. In parallel, HIF-2α was up-regulated, albeit to a much lesser extent, by DKG in both MDA-MB-231 and MCF7 cells (Figs. 2A, 2B), suggesting that DKG likely targets a common pathway governing both HIF-1α and HIF-2α abundance. Comparable results were observed using a hypoxia-mimetic agent, DFO, in both MDA-MB-231 and MCF7 cells (Figs. S1B, S1C).

Furthermore, to evaluate whether DKG has an impact on global protein synthesis, we looked at the surrogate markers for mTOR activation, phospho-p70S6K and phospho-4EBP1 signals. We observed that DKG-treatment led to a decrease of phospho-p70S6K and total 4EBP1 signals (Fig. 2C). Although this observation was consistent with a recent report showing that α-KG blocks mTOR activity through the inhibition of ATP synthase [29], it was unlikely that the down-regulation of mTOR signaling, hence the decreased mTOR-mediated protein synthesis, accounted for the elevated HIF-1α abundance upon DKG-treatment. To further rule out this possibility, we further revealed that DKG was still able to induce HIF-α accumulation, albeit to a lesser extent, upon Gln depletion (Fig. 2C, *lanes 5 and 6* versus *lane 4*), a condition known to suppress HIF-1α translation [17] (Fig. 2C, *lane 4* versus *lane 1*). Based on these observations that DKG increased HIF-1α abundance without increasing *HIF-1α* steady-state mRNA level and without enhancing mTOR signaling, we hypothesized that DKG impaired the degradation of HIF-1α. To test this possibility, MDA-MB-231 cells were pre-treated with MG132, a proteasome inhibitor known to retard proteasome-dependent protein degradation [30], to elevate HIF-1α abundance under normoxia, and then followed by the treatment of DKG or vehicle to assess HIF-1α abundance in the presence of MG132. We found that DKG had no additional effect on HIF-1α level when proteasome-dependent degradation was blocked (Fig. 2D), implicating that DKG and MG132 acted on the same pathway. To understand at which step DKG acted to inhibit HIF-1α degradation, we compared HIF-1α abundance in MDA-MB-231 cells treated with a combination of DKG, dimethyloxalyglycine (DMOG), which inhibits HIF-1α hydroxylation, DFO and MG132. The levels of HIF-1α in cells undergoing different treatment were almost comparable, irrespective of the combination (Fig. 2E), further supporting that DKG, DMOG and DFO may all act in the same pathway to retard HIF-1α degradation. To ascertain this, MDA-MB-231 cells were treated with DKG, DMOG or both and HIF-1α levels were determined. DKG or DMOG alone increased HIF-1α, but combining them did not further elevate HIF-1α abundance, compared to the single treatments (Fig. 2F). We therefore postulated that DKG and DMOG acted redundantly to increase HIF-1α stability. Collectively, these data supported a mechanism in which DKG blocked HIF-1α proteasomal degradation.

### DKG inhibits HIF-1α proline hydroxylation and degradation mediated by PHD2

**Figure 3:**
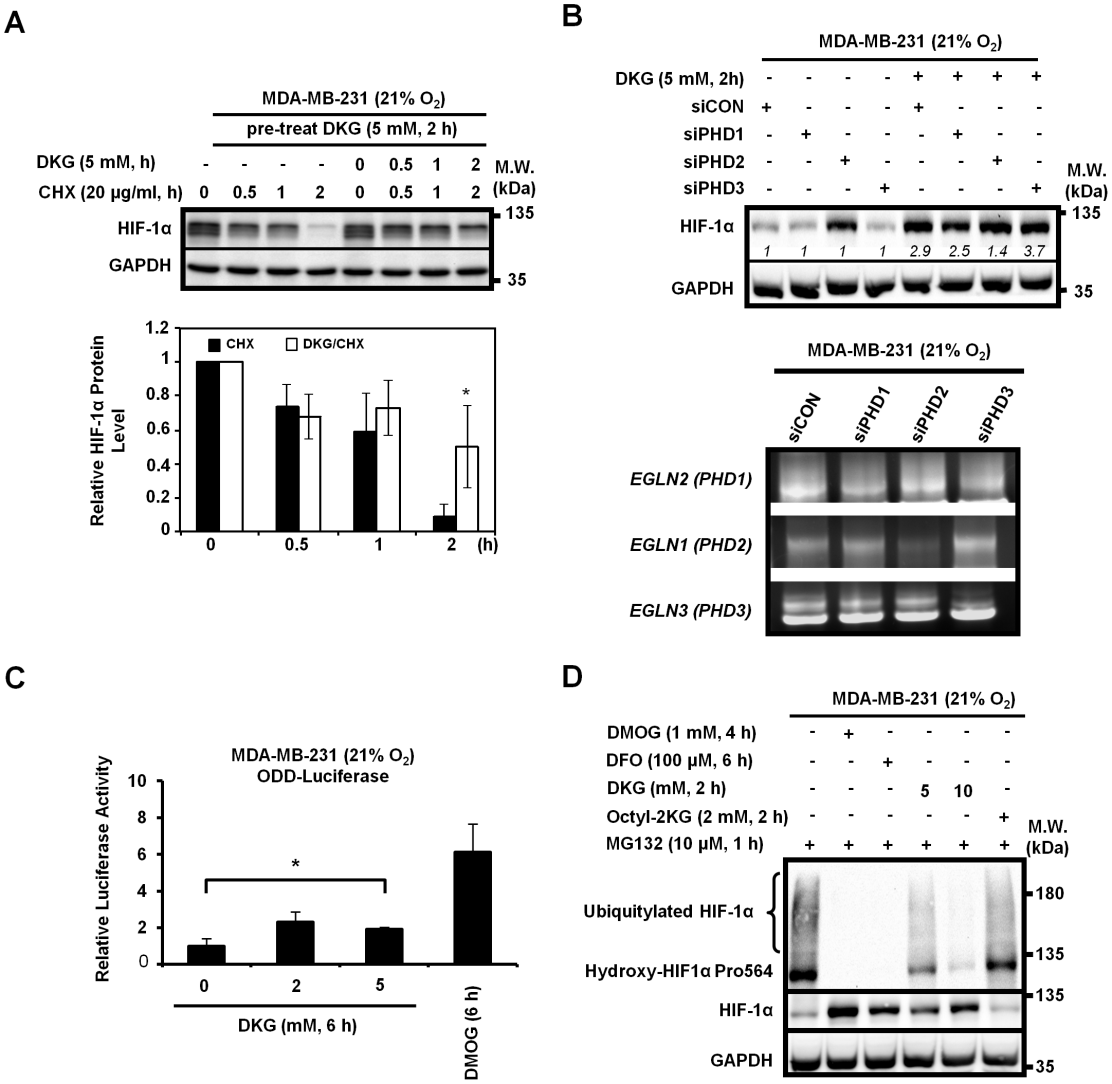
DKG stabilizes HIF-1α by inhibiting HIF-1α proline hydroxylation and degradation. (**A**) DKG increases HIF-1α protein stability. MDA-MB-231 cells were pretreated with DKG (5 mM, 2-hour), then treated with CHX (20 µg/ml) in the presence or absence of DKG (5 mM) for an additional time period as indicated (*upper panel*). The relative abundance of HIF-1α is shown (*lower panel*). _*_: *p*<0.05; n = 3. (**B**) DKG blocks HIF-1α degradation mediated by PHD2. MDA-MB-231 cells were transfected with control siRNA or siRNAs targeting PHD1, PHD2 or PHD3 before treating with DKG (5 mM, 2-hour) (upper panel). *Italic numbers* represent the relative quantitation of protein levels. The level in the untreated sample from each pair of siCON-transfected, untreated cells was set as 1. The extent of respective knockdown of PHD1, 2 or 3 was assessed with semi-quantitative nested RT-PCR, followed by agarose gel electrophoresis (*lower panel*). (**C**) DKG increases HIF-1α stability through its oxygen-dependent degradation domain (ODD). ODD-luciferase activity was assayed in MDA-MB-231 cells co-transfected with ODD-luciferase reporter and control renilla luciferase reporter and treated with increasing doses of DKG for 6-hour. DMOG-treated cells serve a positive control. _*_: *p*<0.05; n = 3. (**D**) Hydroxylation at Pro564 of HIF-1α is inhibited by DKG. MDA-MB-231 cells were treated with the proteasome inhibitor, MG132, combined with the indicated chemicals to assess the level of HIF-1α hydroxylated at Pro564. The high-molecular weight, smeared species are the ubiquitylated HIF-1α. (**A**, **B**, **D**) A representative Western image from 3 independent experiments is shown.

To explore how DKG impairs HIF-1α degradation, we next compared the HIF-1α half-life in cells in the presence and absence of DKG. First, cells were pre-treated with DKG for 2-hour to boost HIF-1α level, then incubated in the presence of cycloheximide (CHX) to block protein biosynthesis and treated or untreated with DKG. The half-life of HIF-1α induced by DKG was between 1- and 2-hour after DKG withdrawal, but continuous exposure to DKG prolonged the half-life of HIF-1α induced by DKG to longer than 2-hour (Fig. 3A). The observation that DKG extended HIF-1α half-life further corroborated that DKG inhibits HIF-1α degradation. Because PHDs use α-KG as a cofactor to catalyze hydroxylation on the substrates, we then tested whether DKG stabilized HIF-1α through modulating the activity of PHDs. Under normoxia, knocking down PHD2, but not PHD1 or 3 (Fig. 3B, *lower panel*), markedly increased HIF-1α abundance (Fig. 3B, *upper panel*). This observation was consistent with the previous reports that HIF-1α stability was primarily regulated by PHD2 [6], [31]. Notably, treatment of DKG did not further induce the accumulation of HIF-1α in the PHD2-knockdown cells, while HIF-α was still up-regulated by DKG in the PHD1 or PHD3-knockdown cells (Fig. 3B, *upper panel*). Taken together, we concluded that DKG promoted HIF-1α accumulation, at least in part, by inhibiting the PHD2-mediated HIF-1α degradation. Moreover, results from ODD-luciferase reporter assays supported that DKG directly inhibited PHD activity on the ODD hydroxylation (Fig. 3C). Lastly, we used a specific antibody that recognized HIF-1α hydroxylated at Pro564 to determine the levels of hydroxylated HIF-1α when proteasomal degradation was inhibited by MG132. Treating the cells with DMOG, completely inhibited both the hydroxylation and ubiquitylation of HIF-1α (Fig. 3D, *lane 2* versus *lane 1*), and served as a control. Likewise, DFO, an iron chelator that modulates PHD activity, blocked the hydroxylation of HIF-1α (Fig. 3D. *lane 3*). We found that DKG reduced the levels of hydroxylated HIF-1α in a dose-dependent manner, whereas Octyl-2KG did not (Fig. 3D). Notably, the total HIF-1α abundances were inversely correlated with the levels of hydroxylated HIF-1α (Fig. 3D, *1^st^ and 2^nd^ panels*). Altogether, we concluded that DKG targeted PHD2 to block the oxygen-dependent hydroxylation of HIF-1α at Pro564, thereby stabilizing HIF-1α.

### DKG enhanced HIF-1α downstream signaling

**Figure 4:**
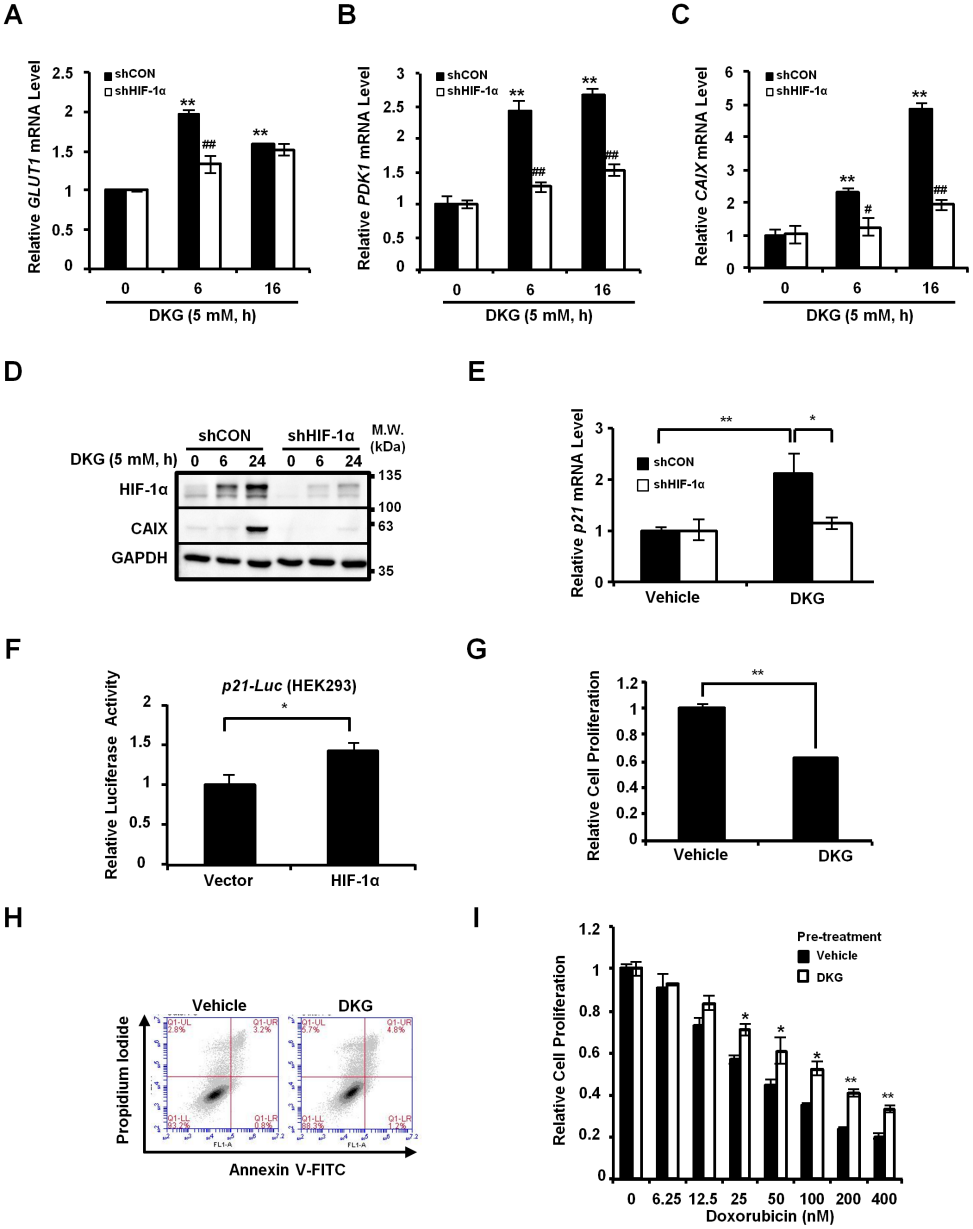
DKG activates HIF-1α downstream signaling. (**A, B, C**) DKG induces HIF-1α target gene expression. *GLUT1 (A)*, *PDK1 (B) and CAIX (C)* mRNA abundance was assessed in MDA-MB-231/shCON and/shHIF-1α cells treated with DKG (5 mM, 6- or 16-hour) by quantitative PCR. _*_: *p*<0.05; _**_: *p*<0.01 (DKG-treated versus. untreated); #: *p*<0.05; ##: *p*<0.01 (shHIF-1α versus. shCON); n = 3. (**D**) CAIX is induced by DKG in a HIF-1α-dpenendent manner. CAIX protein level in the MDA-MB-231/shCON and/shHIF-1α cells treated with DKG (5 mM, 6- or 24-hour) was examined by Western blot analyses. A representative Western image from 3 independent experiments is shown. (**E**) DKG mediates a HIF-1α-dependent increase of *p21* mRNA abundance. *p21* mRNA abundance was measured in MDA-MB-231/shCON and/shHIF-1α cells treated with DKG (10 mM, 48-hour) by quantitative PCR. (**F**) HIF-1α activates *p21-Luc* reporter. *p21-Luc* reporter activity was quantitated by luciferase assays in HEK293 cells co-transfected with a combination of HIF-1α expression construct, *p21-Luc*, and renilla control reporter. (**G**) DKG inhibits cell proliferation. Cell proliferation was determined by measuring the activity of acid phosphatase (ACP) in the MDA-MB-231 cells treated with DKG (10 mM, 72-hour). (**H**) DKG does not induce apoptosis/cell death. Annexin V/PI double staining was performed to quantitate apoptosis/cell death in MDA-MB-231 cells treated with DKG (10 mM, 24-hour). (**I**) DKG pre-treatment renders doxorubicin resistance. MDA-MB-231 cells were pre-treated with DKG (10 mM, 7-day) and then treated with increasing doses of doxorubicin (0–400 nM). Cell proliferation was measured by ACP assay. _*_: *p*<0.05; _**_: *p*<0.01; n = 3.

To determine whether the HIF-1α protein induced by DKG was transcriptionally functional, we assessed several HIF-1α downstream target genes in DKG-treated cells. Steady-state levels of *GLUT1*, *PDK1* and *CAIX* mRNA were significantly up-regulated in DKG-treated MDA-MB-231 cells (Figs. 4A–4C) and the induction of *GLUT1*, *PDK1* and *CAIX* was HIF-1α-dependent as knockdown of HIF-1α by a short hairpin (sh) RNA dampened the abundances of these three messages induced by DKG-treatment (Figs. 4A–4C). CAIX protein level also significantly increased by DKG in a HIF-1α-dependent manner (Fig. 4D). Moreover, HIF-1α was reportedly to up-regulate *p21* expression to arrest cell cycle progression [32]. Consistently, we observed that there was a HIF-1α-dependent up-regulation of *p21* message abundance upon DKG-treatment and the knockdown of HIF-1α reduced *p21* mRNA induction (Fig. 4E). In addition, to further confirm that DKG-mediated HIF-1α accumulation regulates the transcription of *p21*, we overexpressed HIF-1α and found that the activity of *p21* promoter was significantly increased (Fig. 4F). All together, these results suggested that DKG was able to enhance HIF-1α activity and to induce its downstream signaling, implying that DKG may regulate various HIF-1α-dependent biological processes, including cell cycle progression and Glc metabolism. To explore the biological consequence of increased HIF-1α abundance in response to DKG-treatment, MDA-MB-231 cells were treated with DKG for 72-hour and measured cell proliferation and cell death. Clearly, DKG reduced cell proliferation (Fig. 4G), consistent with *p21* induction, possibly due to the elevation of HIF-1α signaling, as previously described [32], [33]. However, the reduced cell proliferation was not resulted from massive cell death (Fig. 4H). Moreover, pre-treatment of DKG rendered the MDA-MB-231 cells more resistant to a genotoxic agent, doxorubicin (Fig. 4I), corroborating the role of HIF-1α activation in doxorubicin resistance [34].

## Discussion

Conventional wisdom suggests that α-KG is not only a metabolite, but also a co-substrate for a large family of dioxygenases, including PHD family members [35]. HIF-1α is modified by PHDs, and thereafter degraded by proteasome via VHL protein-dependent ubiquitylation [36], [37]. The cell membrane-permeable α-KG precursor, DKG, has been experimentally tested as supplements to rescue cell growth under Gln-deprivation [38], [39]. However, another kind of α-KG analogues, such as Octyl-2KG and related derivatives, significantly down-regulate HIF-1α in multiple cell types, including cancer cells with IDH1 or SDH mutations, by acting as a PHD cofactor to promote PHD function [14], [27]. Counter-intuitively, we show here that DKG stabilized HIF-1α by inhibiting PHD2, thereby creating pseudohypoxia under normoxia and nutrient-rich condition. Conceivably, it is prudent to suggest the possibility that DKG possesses a previously unrecognized non-canonical function to promote HIF-1α accumulation under nutrient-rich condition, in addition to serving as its canonical function of rescuing cell proliferation under nutrient-deprived condition.

Rzeski et al. have shown that α-KG has anti-proliferative effects on colon cancer cells, that are accompanied by the up-regulation of p21, p27 and down-regulation of cyclin D1 [33]. Furthermore, α-KG can extend the lifespan of *C. elegans*, suggesting that α-KG regulates multiple cellular processes that are important for maintaining the viability of organisms. Our data suggest that DKG creates a pseudohypoxic state by up-regulating HIF-1α. Unlike α-KG or Octyl-2KG, DKG inhibits the proline hydroxylation of HIF-1α by PHD2 (Fig. 3). Given that PHD2 requires α-KG as a cofactor for function, we propose that DKG may be by itself or metabolized or processed (to other than α-KG) to inhibit PHD2 activity. For example, succinate and fumarate are the metabolites downstream of α-KG in TCA cycle, and both of them can inhibit PHD2 activity, either through competing with α-KG binding to PHD2, or by intercalating with glutathione to induce the production of reactive oxygen species (ROS) [12], [13], [40]. We suspect that DKG could be rapidly processed to succinate or fumarate under nutrient-rich conditions, thereby inhibiting PHD2. Another possibility is that the intracellular ROS levels, elevated under pseudohypoxia induced by DKG, lead to the HIF-1α stabilization. Increased ROS has also been linked to PHD2 inhibition and HIF-1α accumulation [41], [42]. Indeed, the accumulation of HIF-1α induced by DKG was partially suppressed by pre-treating cells with N-acetylcysteine, a ROS scavenger (data not shown).

Under normoxic conditions, HIF-1α is efficiently degraded. PHD2 is the major regulator controlling the degradation of HIF-1α (Fig. 3B) [6], [31]. The regulation is complicated by the hypoxic induction of PHD2 and PHD3, and additional mechanisms to promote HIF accumulation may be required to prevent complete HIF degradation in the chronic hypoxic conditions found in tumors [43]. Of note, PHDs are not the only biological means for α-KG to inactivate HIF-1α [4]. For example, the oxygen-dependent hydroxylase factor inhibiting HIF hydroxylates asparagine (Asn803) residue in the C-terminal transactivation domain of HIF-1α in an α-KG-dependent manner, reducing the binding of HIF-1α to transcriptional co-activators [44], [45]. Additional regulatory modifications of the HIF-1α include SUMOylation [46], [47], acetylation [48] and phosphorylation [49], [50]. Although the effect of DKG on these HIF-1α post-translational modifications other than PHD2-dependent proline-hydroxylation and ubiquitylation remains to be investigated, it is clear that DKG activates HIF-1α signaling (Fig. 4).

In summary, our study identifies DKG as a potent activator of HIF-1α by stabilizing HIF-1α in a PHD2-dependent manner under both normoxia and hypoxia. HIF-1α is known to play a major role in tumorigenesis, through activation of several genes implicated in many aspects of cancer progression and prognosis [2]. On the other hand, PHD2 has been shown to be down-regulated in human breast cancers [51]. Based on our results, we show that inhibition of PHD2 by DKG increases the expression of genes implicated in the glycolytic pathway (*GLUT1* and *PDK1*), the mechanism known to play an important role in cancer progression. Hence, DKG could be a useful molecule allowing us to investigate DKG-PHD2-dependent regulation of specific HIF-1α target genes in order to better understand hypoxia signaling mechanisms and for the identification of new therapeutic targets.

## Supporting Information

Figure S1 (**A**) **DKG does not up-regulate** ***HIF-1α*** **mRNA abundance.** Quantitative PCR was used to assess *HIF-1α* mRNA levels in MDA-MB-231 cells treated either with DKG (10 mM) for the indicated time periods, or DFO (100 µM, 8-hour) under normoxia; n = 3. (**B, C**) DKG induces HIF-1α in both normoxic and DFO-mimicked hypoxic conditions. MDA-MB-231 (B) and MCF7 (C) cells were treated with DFO (100 µM) for 2-, 6- or 24-hour in the presence or absence of DKG (5 mM). *Italic numbers* indicate the relative protein level after normalization with the level in the untreated cells.
(TIF)

## References

[1] Semenza GL (2014) Oxygen Sensing, Hypoxia-Inducible Factors, and Disease Pathophysiology. Annual Review of Pathology: Mechanisms of Disease 9:47–71. 10.1146/annurev-pathol-012513-104720 23937437

[2] Semenza GL (2009) Defining the role of hypoxia-inducible factor 1 in cancer biology and therapeutics. Oncogene 29:625–634. 19946328 10.1038/onc.2009.441 PMC2969168

[3] Schofield CJ, Ratcliffe PJ (2004) Oxygen sensing by HIF hydroxylases. Nat Rev Mol Cell Biol 5:343–354. 15122348 10.1038/nrm1366

[4] Kaelin Jr WG, Ratcliffe PJ (2008) Oxygen Sensing by Metazoans: The Central Role of the HIF Hydroxylase Pathway. Molecular Cell 30:393–402. 18498744 10.1016/j.molcel.2008.04.009

[5] Jaakkola P, Mole DR, Tian Y-M, Wilson MI, Gielbert J, et al (2001) Targeting of HIF-α to the von Hippel-Lindau Ubiquitylation Complex by O2-Regulated Prolyl Hydroxylation. Science 292:468–472. 11292861 10.1126/science.1059796

[6] Berra E, Benizri E, Ginouvès A, Volmat V, Roux D, et al (2003) HIF prolyl-hydroxylase 2 is the key oxygen sensor setting low steady-state levels of HIF-1α in normoxia. EMBO J 22:4082–4090. 12912907 10.1093/emboj/cdg392 PMC175782

[7] Bubendorf L, Nocito A, Moch H, Sauter G (2001) Tissue microarray (TMA) technology: miniaturized pathology archives for high-throughput in situ studies. The Journal of Pathology 195:72–79. 11568893 10.1002/path.893

[8] Chan DA, Sutphin PD, Yen S-E, Giaccia AJ (2005) Coordinate Regulation of the Oxygen-Dependent Degradation Domains of Hypoxia-Inducible Factor 1α. Molecular and Cellular Biology 25:6415–6426. 16024780 10.1128/MCB.25.15.6415-6426.2005 PMC1190339

[9] Masson N, Willam C, Maxwell PH, Pugh CW, Ratcliffe PJ (2001) Independent function of two destruction domains in hypoxia-inducible factor-α chains activated by prolyl hydroxylation. 5197–5206 p. 10.1093/emboj/20.18.5197 PMC125617 11566883

[10] Semenza GL (2012) Hypoxia-Inducible Factors in Physiology and Medicine. Cell 148:399–408. 22304911 10.1016/j.cell.2012.01.021 PMC3437543

[11] Pugh CW, Ratcliffe PJ (2003) The von Hippel–Lindau tumor suppressor, hypoxia-inducible factor-1 (HIF-1) degradation, and cancer pathogenesis. Seminars in Cancer Biology 13:83–89. 12507560 10.1016/s1044-579x(02)00103-7

[12] Isaacs JS, Jung YJ, Mole DR, Lee S, Torres-Cabala C, et al (2005) HIF overexpression correlates with biallelic loss of fumarate hydratase in renal cancer: Novel role of fumarate in regulation of HIF stability. Cancer Cell 8:143–153. 16098467 10.1016/j.ccr.2005.06.017

[13] Selak MA, Armour SM, MacKenzie ED, Boulahbel H, Watson DG, et al (2005) Succinate links TCA cycle dysfunction to oncogenesis by inhibiting HIF-α prolyl hydroxylase. Cancer Cell 7:77–85. 15652751 10.1016/j.ccr.2004.11.022

[14] Zhao S, Lin Y, Xu W, Jiang W, Zha Z, et al (2009) Glioma-Derived Mutations in IDH1 Dominantly Inhibit IDH1 Catalytic Activity and Induce HIF-1α. Science 324:261–265. 19359588 10.1126/science.1170944 PMC3251015

[15] Del Bufalo D, Ciuffreda L, Trisciuoglio D, Desideri M, Cognetti F, et al (2006) Antiangiogenic Potential of the Mammalian Target of Rapamycin Inhibitor Temsirolimus. Cancer Research 66:5549–5554. 16740688 10.1158/0008-5472.CAN-05-2825

[16] Luo W, Zhong J, Chang R, Hu H, Pandey A, et al (2010) Hsp70 and CHIP Selectively Mediate Ubiquitination and Degradation of Hypoxia-inducible Factor (HIF)-1α but Not HIF-2α. Journal of Biological Chemistry 285:3651–3663. 19940151 10.1074/jbc.M109.068577 PMC2823506

[17] Kwon SJ, Lee YJ (2005) Effect of Low Glutamine/Glucose on Hypoxia-Induced Elevation of Hypoxia-Inducible Factor-1α in Human Pancreatic Cancer MiaPaCa-2 and Human Prostatic Cancer DU-145 Cells. Clinical Cancer Research 11:4694–4700. 16000563 10.1158/1078-0432.CCR-04-2530

[18] Roecklein B, Torok-Storb B (1995) Functionally distinct human marrow stromal cell lines immortalized by transduction with the human papilloma virus E6/E7 genes. Blood 85:997–1005. 7849321

[19] Tait L, Soule HD, Russo J (1990) Ultrastructural and Immunocytochemical Characterization of an Immortalized Human Breast Epithelial Cell Line, MCF-10. Cancer Research 50:6087–6094. 1697506

[20] Clavijo C, Chen C, Kim K-J, Reyland ME, Ann DK (2007) Protein kinase Cδ-dependent and -independent signaling in genotoxic response to treatment of desferroxamine, a hypoxia-mimetic agent. American Journal of Physiology - Cell Physiolog 292:C2150–C2160. 10.1152/ajpcell.00425.2006 17563398

[21] Chen J-L, Lin HH, Kim K-J, Lin A, Forman HJ, et al (2008) Novel Roles for Protein Kinase Cδ-dependent Signaling Pathways in Acute Hypoxic Stress-induced Autophagy. Journal of Biological Chemistry 283:34432–34444. 18836180 10.1074/jbc.M804239200 PMC2590682

[22] Lee Y-K, Thomas SN, Yang AJ, Ann DK (2007) Doxorubicin Down-regulates Kruppel-associated Box Domain-associated Protein 1 Sumoylation That Relieves Its Transcription Repression on p21WAF1/CIP1 in Breast Cancer MCF-7 Cells. J Biol Chem 282:1595–1606. 17079232 10.1074/jbc.M606306200

[23] Li X, Lee YK, Jeng JC, Yen Y, Schultz DC, et al (2007) Role for KAP1 serine 824 phosphorylation and sumoylation/desumoylation switch in regulating KAP1-mediated transcriptional repression. J Biol Chem 282:36177–36189. 17942393 10.1074/jbc.M706912200

[24] Kondo K, Klco J, Nakamura E, Lechpammer M, Kaelin Jr WG (2002) Inhibition of HIF is necessary for tumor suppression by the von Hippel-Lindau protein. Cancer Cell 1:237–246. 12086860 10.1016/s1535-6108(02)00043-0

[25] Safran M, Kim WY, O'Connell F, Flippin L, Günzler V, et al (2006) Mouse model for noninvasive imaging of HIF prolyl hydroxylase activity: Assessment of an oral agent that stimulates erythropoietin production. Proceedings of the National Academy of Sciences of the United States of America 103:105–110. 16373502 10.1073/pnas.0509459103 PMC1324998

[26] Wang GL, Semenza GL (1993) Desferrioxamine induces erythropoietin gene expression and hypoxia- inducible factor 1 DNA-binding activity: implications for models of hypoxia signal transduction. Blood 82:3610–3615. 8260699

[27] MacKenzie ED, Selak MA, Tennant DA, Payne LJ, Crosby S, et al (2007) Cell-Permeating α-Ketoglutarate Derivatives Alleviate Pseudohypoxia in Succinate Dehydrogenase-Deficient Cells. Molecular and Cellular Biology 27:3282–3289. 17325041 10.1128/MCB.01927-06 PMC1899954

[28] Carroll VA, Ashcroft M (2006) Role of Hypoxia-Inducible Factor (HIF)-1α versus HIF-2α in the Regulation of HIF Target Genes in Response to Hypoxia, Insulin-Like Growth Factor-I, or Loss of von Hippel-Lindau Function: Implications for Targeting the HIF Pathway. Cancer Research 66:6264–6270. 16778202 10.1158/0008-5472.CAN-05-2519

[29] Chin R, Fu X, Pai M, Vergnes L, Hwang H, et al (2014) The metabolite alpha-ketoglutarate extends lifespan by inhibiting ATP synthase and TOR. Nature 510:397–401. 24828042 10.1038/nature13264 PMC4263271

[30] Lee DH, Goldberg AL (1998) Proteasome inhibitors: valuable new tools for cell biologists. Trends in Cell Biology 8:397–403. 9789328 10.1016/s0962-8924(98)01346-4

[31] Tennant DA, Frezza C, MacKenzie ED, Nguyen QD, Zheng L, et al (2009) Reactivating HIF prolyl hydroxylases under hypoxia results in metabolic catastrophe and cell death. Oncogene 28:4009–4021. 19718054 10.1038/onc.2009.250

[32] Koshiji M, Kageyama Y, Pete EA, Horikawa I, Barrett JC, et al (2004) HIF-1[alpha] induces cell cycle arrest by functionally counteracting Myc. EMBO J 23:1949–1956. 15071503 10.1038/sj.emboj.7600196 PMC404317

[33] Rzeski W, Walczak K, Juszczak M, Langner E, PoŻarowski P, et al (2012) Alpha-ketoglutarate (AKG) inhibits proliferation of colon adenocarcinoma cells in normoxic conditions. Scandinavian Journal of Gastroenterology 47:565–571. 22486188 10.3109/00365521.2012.660539

[34] Song X, Liu X, Chi W, Liu Y, Wei L, et al (2006) Hypoxia-induced resistance to cisplatin and doxorubicin in non-small cell lung cancer is inhibited by silencing of HIF-1α gene. Cancer Chemotherapy and Pharmacology 58:776–784. 16532342 10.1007/s00280-006-0224-7

[35] Loenarz C, Schofield CJ (2008) Expanding chemical biology of 2-oxoglutarate oxygenases. Nat Chem Biol 4:152–156. 18277970 10.1038/nchembio0308-152

[36] Epstein ACR, Gleadle JM, McNeill LA, Hewitson KS, O'Rourke J, et al (2001) C. elegans EGL-9 and Mammalian Homologs Define a Family of Dioxygenases that Regulate HIF by Prolyl Hydroxylation. Cell 107:43–54. 11595184 10.1016/s0092-8674(01)00507-4

[37] Zhang Y, Shao Z, Zhai Z, Shen C, Powell-Coffman JA (2009) The HIF-1 Hypoxia-Inducible Factor Modulates Lifespan in C. elegans. PLoS ONE 4:e6348. 19633713 10.1371/journal.pone.0006348 PMC2711329

[38] Wise DR, Ward PS, Shay JES, Cross JR, Gruber JJ, et al (2011) Hypoxia promotes isocitrate dehydrogenase-dependent carboxylation of α-ketoglutarate to citrate to support cell growth and viability. Proceedings of the National Academy of Sciences 108:19611–19616. 10.1073/pnas.1117773108 PMC3241793 22106302

[39] Lin T-C, Chen Y-R, Kensicki E, Li AY-J, Kong M, et al (2012) Autophagy: Resetting glutamine-dependent metabolism and oxygen consumption. Autophagy 8:1477–1493. 22906967 10.4161/auto.21228 PMC3679231

[40] Sullivan Lucas B, Martinez-Garcia E, Nguyen H, Mullen Andrew R, Dufour E, et al (2013) The Proto-oncometabolite Fumarate Binds Glutathione to Amplify ROS-Dependent Signaling. Molecular cell 51:236–248. 23747014 10.1016/j.molcel.2013.05.003 PMC3775267

[41] Niecknig H, Tug S, Reyes BD, Kirsch M, Fandrey J, et al (2012) Role of reactive oxygen species in the regulation of HIF-1 by prolyl hydroxylase 2 under mild hypoxia. Free Radical Research 46:705–717. 22360728 10.3109/10715762.2012.669041

[42] Guzy RD, Hoyos B, Robin E, Chen H, Liu L, et al (2005) Mitochondrial complex III is required for hypoxia-induced ROS production and cellular oxygen sensing. Cell Metabolism 1:401–408. 16054089 10.1016/j.cmet.2005.05.001

[43] Appelhoff RJ, Tian Y-M, Raval RR, Turley H, Harris AL, et al (2004) Differential Function of the Prolyl Hydroxylases PHD1, PHD2, and PHD3 in the Regulation of Hypoxia-inducible Factor. Journal of Biological Chemistry 279:38458–38465. 15247232 10.1074/jbc.M406026200

[44] Mahon PC, Hirota K, Semenza GL (2001) FIH-1: a novel protein that interacts with HIF-1α and VHL to mediate repression of HIF-1 transcriptional activity. Genes & Development 15:2675–2686. 11641274 10.1101/gad.924501 PMC312814

[45] **Zhang N, Fu Z, Linke S, Chicher J, Gorman JJ, et al.** The Asparaginyl Hydroxylase Factor Inhibiting HIF-1α Is an Essential Regulator of Metabolism. Cell Metabolism 11:364–378. 20399150 10.1016/j.cmet.2010.03.001 PMC2893150

[46] Cheng J, Kang X, Zhang S, Yeh ETH (2007) SUMO-Specific Protease 1 Is Essential for Stabilization of HIF1α during Hypoxia. Cell 131:584–595. 17981124 10.1016/j.cell.2007.08.045 PMC2128732

[47] Kang X, Li J, Zou Y, Yi J, Zhang H, et al (2010) PIASy stimulates HIF1[alpha] SUMOylation and negatively regulates HIF1[alpha] activity in response to hypoxia. Oncogene 29:5568–5578. 20661221 10.1038/onc.2010.297

[48] Jeong J-W, Bae M-K, Ahn M-Y, Kim S-H, Sohn T-K, et al (2002) Regulation and Destabilization of HIF-1α by ARD1-Mediated Acetylation. Cell 111:709–720. 12464182 10.1016/s0092-8674(02)01085-1

[49] Flügel D, Görlach A, Michiels C, Kietzmann T (2007) Glycogen Synthase Kinase 3 Phosphorylates Hypoxia-Inducible Factor 1α and Mediates Its Destabilization in a VHL-Independent Manner. Molecular and Cellular Biology 27:3253–3265. 17325032 10.1128/MCB.00015-07 PMC1899978

[50] Warfel NA, Dolloff NG, Dicker DT, Malysz J, El-Deiry WS (2013) CDK1 stabilizes HIF-1α via direct phosphorylation of Ser668 to promote tumor growth. Cell Cycle 12:3689–3701. 24189531 10.4161/cc.26930 PMC3903720

[51] Bordoli MR, Stiehl DP, Borsig L, Kristiansen G, Hausladen S, et al (2011) Prolyl-4-hydroxylase PHD2- and hypoxia-inducible factor 2-dependent regulation of amphiregulin contributes to breast tumorigenesis. Oncogene 30:548–560. 20856199 10.1038/onc.2010.433

